# Indole Derivatives: Unveiling New Frontiers in Medicinal and Synthetic Organic Chemistry

**DOI:** 10.3390/molecules28145477

**Published:** 2023-07-18

**Authors:** Faiza Saleem, Khalid Mohammed Khan

**Affiliations:** 1 Husein Ebrahim Jamal Research Institute of Chemistry, International Center for Chemical and Biological Sciences, University of Karachi, Karachi 75270, Pakistan; faizasaleem632@gmail.com; 2 Pakistan Academy of Science, 3-Constitution Avenue, G-5/2, Islamabad 44000, Pakistan

In recent years, significant attention has been given to indoles, a diverse group of heterocyclic compounds widely found in nature that play a crucial role in various bioactive natural and synthetic substances [1]. The synthesis of diverse indole-containing compounds is fascinating due to their significance as intermediates in antimicrobial and antiviral compounds and as building blocks for bioactive molecules [2]. Consequently, there is a growing interest in conducting scientific research to explore the synthesis, bioactivities, and potential effects of novel indole compounds, which hold great promise for various practical applications. Given the widespread interest in this field, we aim to continue gathering unique and compelling research studies focusing on indole compounds and their biological effects.

Four review papers are in the current Special Issue. A detailed review was carried out to investigate the potential of target-driven anticancer indole derivatives for developing highly effective agents against glioblastoma (GBM), the most aggressive form of primary brain tumor. Current GBM therapy involves surgery, radiotherapy, and chemotherapy, but their modest efficacy highlights the urgent need for novel therapies. Indole, known for its significance in heterocyclic chemistry, holds promise as a platform for developing new drug candidates to combat GBM. The review extensively examined the therapeutic advantages of innovative indole-based derivatives explored as potential anti-GBM agents. The main focus was evaluating the compounds currently undergoing preclinical and clinical development for GBM, with a notable emphasis on advances from 2013 to 2022. The review also covered the fundamental mechanisms of action, including the inhibition of protein kinase, tubulin, and the p53 pathway, which play a critical role in their anti-GBM activity. The goal is to inspire medicinal chemists to design and develop effective indole-based GBM treatment agents [3].

A featured review article on indole-containing natural products demonstrated that indole natural products, particularly indole alkaloids, have gained significant attention for their diverse biological activities. It summarizes recent findings on 250 novel indole alkaloids, including their isolation, characterization, and biological potential. Moreover, the review offers a reevaluation of previously reported compounds and investigates the complete synthesis of indole alkaloids. Additionally, it delves into the synthesis and semi-synthesis of derivatives containing indole and examines their reported bioactivities during 2019–2022. Compiling this information is valuable for researchers, as it facilitates further exploration and development of indole-based compounds for therapeutic applications [2]. Another article featured in this Special Issue explores the significance of spirooxindoles, which have gained attention due to their appealing structure and wide range of pharmacological properties, making them attractive scaffolds. Recent years have witnessed several investigations on spirooxindole scaffolds, which have been thoroughly described. Collecting the synthetic protocols for pharmacologically active spirooxindole scaffolds is an efficient foundation for developing new spirooxindole compounds targeting various diseases [4]. Another article presents a detailed analysis of kinase inhibitors, emphasizing those possessing indole, azaindole, or oxindole scaffolds. It highlights that thirty of these inhibitors have been approved for therapeutic purposes in various diseases, while three are currently undergoing clinical trials and one is in the preliminary stages of preclinical studies. The process of discovering and optimizing these approved inhibitors and their mutant drug routes is described. The authors investigate the bonding interactions between the inhibitors and specific amino acid residues in the ATP-binding sites of target kinases. Their findings reveal that the studied frameworks are vital in establishing bonds within the hinge region of the kinase’s ATP-binding site. This interaction enhances the inhibitors’ ability to bind effectively to the ATP pocket. The research findings indicate that these heterocyclic systems possess privileged scaffolds for developing new ATP-competitive kinase inhibitors. The authors hope that this review, combined with AI technology, will aid researchers in developing potent and selective kinase inhibitors quickly, given their current prominence as a preferred treatment option for many diseases [5]. A new synthetic approach has been devised to construct the pentacyclic scaffold in the aromathecin family. The strategy involves the sequential synthesis of the isoquinolone and indolizidine moieties. In the process, the crucial step is the thermal cyclization of 2-alkynyl benzaldehyde oxime, which produces the isoquinoline *N*-oxide. Subsequently, this *N*-oxide undergoes a Reissert-Henze-type reaction. Through the optimization of reaction conditions, such as the application of microwave irradiation-assisted heating, the desired isoquinolone is obtained with a high yield. Applying this approach, the simplest member of the aromathecin family, rosettacin, was synthesized with a noteworthy overall yield. Moreover, the newly developed methodology can potentially synthesize analogs of rosettacin and other fused indolizidine compounds [6].

Researchers conducted a study to discover new bioactive compounds by combining molecules with biological properties to produce indole derivatives. Ten (10) derivatives were synthesized, featuring different moieties at the C-3 position. The compounds were characterized for their antibacterial, antioxidant, and fungicidal activities. The study also involved determining five compounds’ crystal structures, revealing the impact of ring atom substitutions on molecular conformation, intermolecular interactions, and biological activity. Indole-imidazole derivatives with alkyl substituents exhibited strong cytoprotective effects and effective chelation of ferrous ions. A chlorine-containing indole-imidazole showed antifungal properties against specific strains, while the derivatives displayed potent antibacterial activity, particularly against *M. luteus* and *P. fluorescens*. The research findings highlight the potential of selective indole derivatives as potent antioxidants and antimicrobial agents [7]. In another study, seven indole-aryl amide derivatives incorporating tryptamine or indolyl acetic acid structures were designed, synthesized, and assessed as opioid ligands. However, these novel indole derivatives displayed minimal to extremely low affinity towards μ- and δ-opioid receptors.

Nonetheless, indoles are widely recognized for their anticancer properties; the compounds were also tested for their effectiveness against various tumor cell lines. Some synthetic compounds demonstrated good activity against the selected tumor cells, except for IGROV1. Compound *N*-(4-aminobenzyl)-*N*-(4-hydroxybenzyl)-2-(1*H*-indol-3-yl) acetamide, in particular, demonstrated substantial selectivity towards HT29 cells, a malignant colonic cell line, while sparing healthy human intestinal cells. Subsequent investigations revealed that this compound effectively induced cell cycle arrest in the G1 phase and facilitated apoptosis in HT29 cells [1].

A study focused on synthesizing new bis-indole derivatives featuring a phenyl linker obtained from indole phytoalexins. Various synthetic methods were used to obtain different bis-indole compounds. Among them, compound *N,N*′-(1,4-phenylene)bis{*N*′-[1-(tert-butoxycarbonyl) indol-3-yl]methyl (urea)} showed promising activity against lung cancer cells (A549) while sparing non-cancerous cells. It also induced autophagy, depleted glutathione, and enhanced the antiproliferative impact of cis-platin. The study highlights the compound’s potential as a therapeutic agent for lung cancer treatment [8].

The study conducted by Kim et al. focuses on the synthesis of innovative 1-acyloxy indole compounds and investigates the pathways involved in their reactions. The starting material, a nitro ketoester substrate, undergoes a series of transformations, including reduction, intramolecular addition, nucleophilic 1,5-addition, and acylation, in a single reaction vessel, leading to the formation of 1-acyloxy indoles. By conducting systematic studies, they establish optimized reaction conditions, focusing on the final acylation step of the intermediate 1-hydroxy indole. Under these optimized conditions, they successfully synthesized twenty-one (21) new examples of 1-acyloxy indole derivatives, albeit with modest yields ranging from 24 to 35%. The 1-acetoxy indole compounds exhibit lower stability and yields than other 1-acyloxy indoles. Remarkably, the one-pot reactions involving a four-step sequence enable the efficient synthesis of these new compounds, which might otherwise be challenging to obtain [9]. New indole-containing hybrids derived from millepachine were synthesized and subjected to evaluation. Among them, compound **A** (5-methoxy-2,2-dimethyl-2*H*-chromen-8-yl)(1-methyl-1*H*-indol-5-yl)methanone demonstrated potent cytotoxicity against multiple human cancer cell lines, outperforming millepachine by nearly 100 times. SAR was determined, and mechanistic investigations revealed that compound **A** caused cell-cycle arrest, specifically at the G2/M phase, inhibited tubulin polymerization, and induced cell apoptosis by accumulating reactive oxygen species and collapsing mitochondrial membrane potential. Importantly, compound **A** exhibited low toxicity to normal cells and showed effectiveness against drug-resistant cells, suggesting its potential for developing antitumor drugs [10]. The research primarily focused on *Hypsizygus marmoreus* (*H. marmoreus*) and explored its indole compounds and other bioactive constituents. The study analyzed both commercial and self-cultivated fruiting bodies and the mycelium obtained from in vitro cultures of white and brown varieties. The impact of incorporating zinc and magnesium ions into the culture medium was examined in terms of its influence on the content of the compound. Various analysis techniques were used to determine the composition. The outcomes revealed that mycelium derived from in vitro cultures of *H. marmoreus* is a valuable and abundant source of indole compounds, glucans, bioelements, and lovastatin. Different indole compounds were identified, and enriching the cultures with zinc and magnesium salts increased the levels of certain compounds and elements. The composition varied depending on the *H. marmoreus* variety and the specific material tested [11]. A biotransformation approach was explored to generate acyloins with various indole substituents. The biotransformation system consisted of PfTrpB6, a variant of the standalone β-subunit of tryptophan synthase generated through directed evolution, along with a commercially available L-amino acid oxidase (LAAO) and the ThDP-dependent enzyme NzsH from the neocarazostatin A biosynthetic gene cluster. The PfTrpB variant and LAAO were utilized to produce diverse indole-3-pyruvate derivatives, which served as donor substrates for the NzsH-catalyzed biotransformation to obtain acyloin derivatives. The study demonstrated that NzsH exhibited a broad substrate profile, enabling the production of acyloins with different indole ring systems. These findings suggest the potential of NzsH as a biocatalyst, which could be further improved through directed evolution to enhance its catalytic efficiency in future studies [12]. Serotonin receptors play a role in various physiological functions and behaviors. Three indole derivatives, D2AAK5, D2AAK6, and D2AAK7, were studied as ligands for serotonin 5-HT_1A_ and 5-HT_2A_ receptors. X-ray analysis revealed the crystal structure of D2AAK5, and the main interaction between the ligands and receptors was a salt bridge. Molecular dynamic simulations showed stable ligand-receptor complexes. Remarkably, D2AAK7 exhibited anxiolytic activity, while D2AAK5 positively affected memory processes. These findings highlight the potential of indole derivatives as ligands for serotonin receptors with specific behavioral effects [13]. The study investigated the crystallization behavior of amorphous indomethacin powders under different storage conditions. It found that the *γ*-form polymorph was dominant, but the α-form was accelerated under conditions of higher temperature, increased mobility, and mechanical defects. Crystallization exhibited two distinct growth modes: mechanical defect-induced and free surface-induced. Long-term storage at 10 °C and zero humidity resulted in diffusionless glass-crystal growth, forming a surface crystalline layer of the γ-form. However, due to the abundance of mechanical defects, GC growth was suppressed in fine powders at laboratory temperatures and humidity. Bulk materials without mechanical damage showed no GC growth even after extended storage. The kinetic predictions for indomethacin crystallization were limited due to complex factors such as mechanical defects, competing nucleation, crystal growth processes, and the dependence of GC growth on storage conditions near the glass transition temperature. The study emphasizes the importance of conducting isothermal and nonisothermal measurements in comprehensive studies of drugs with complex crystal growth behaviors [14].

In conclusion, the current Special Issue on indole compounds encompasses many research papers exploring indole derivatives’ synthesis, biological activities, and therapeutic potential. The studies cover diverse areas such as the development of anti-glioblastoma agents, the significance of indole-containing natural products, the exploration of spirooxindoles as pharmacological scaffolds, the role of indole scaffolds in kinase inhibitors, novel synthetic approaches for indole-containing compounds, and the biological properties of indole derivatives. These findings contribute to our understanding of indole chemistry, highlight the potential of indole compounds as valuable sources for drug development, and provide insights for further research and discovery in this field.
